# Determining Repulsion in Cyclophane Cages

**DOI:** 10.3390/molecules27133969

**Published:** 2022-06-21

**Authors:** Mirosław Jabłoński

**Affiliations:** Faculty of Chemistry, Nicolaus Copernicus University in Toruń, 7-Gagarina St., 87-100 Toruń, Poland; teojab@chem.umk.pl; Tel.: +48-056-611-4695

**Keywords:** cage, endohedral complex, exodohedral complex, cyclophane, superphane, repulsion

## Abstract

Superphane, i.e., [2.2.2.2.2.2](1,2,3,4,5,6)cyclophane, is a very convenient molecule in studying the nature of guest⋯host interactions in endohedral complexes. Nevertheless, the presence of as many as six ethylene bridges in the superphane molecule makes it practically impossible for the trapped entity to escape out of the superphane cage. Thus, in this article, I have implemented the idea of using the superphane derivatives with a reduced number of ethylene linkers, which leads to the [2${}_{n}$] cyclophanes where n<6. Seven such cyclophanes are then allowed to form endohedral complexes with noble gas (Ng) atoms (He, Ne, Ar, Kr). It is shown that in the vast majority of cases, the initially trapped Ng atom spontaneously escapes from the cyclophane cage, creating an exohedral complex. This is the best proof that the Ng⋯cyclophane interaction in endohedral complexes is indeed highly repulsive, i.e., destabilizing. Apart from the ‘sealed’ superphane molecule, endohedral complexes are only formed in the case of the smallest He atom. However, it has been shown that in these cases, the Ng⋯cyclophane interaction inside the cyclophane cage is nonbonding, i.e., repulsive. This highly energetically unfavorable effect causes the cyclophane molecule to ‘swell’.

## 1. Introduction

One of the most important goals of chemistry is the identification and description of chemical bonds and various types of long-range intra- and intermolecular interactions. For this reason, it has become very attractive to find the rule given by Bader and based on his Quantum Theory of Atoms in Molecules (QTAIM) [1,2,3] that the simultaneous presence of a bond path (BP) and a bond critical point (BCP) between any pair of atoms is a necessary and sufficient condition for these atoms to be bonded together [4]. Indeed, in many cases (albeit rather simple), the molecular graphs (i.e., the arrangement of bond paths and critical points) obtained with QTAIM coincide with the distribution of bonds on the structural formulas of molecules [5]. Thus, the rule has become an important tool in the hands of chemists to search for chemical bonds and interactions (e.g., hydrogen bonds), and still quite often serves as evidence of their presence.

However, already in the 1990s, Cioslowski began to suggest that the simultaneous presence of BP and BCP does not necessarily prove the presence of a stabilizing interaction, as BP and BCP may also occur for destabilizing, i.e., repulsive interactions [6,7,8,9]. This would be the case especially for highly sterically crowded systems. For this reason, endohedral complexes, i.e., those being composed of a host molecule having a cage structure and a guest entity (atom, ion, or small molecule) trapped therein, have become particularly attractive [10,11,12,13,14]. For example, Haaland et al. [11,12] have shown that the interaction between the encapsulated helium atom and the adamantane cage in the He@adamantane endohedral complex is actually destabilizing despite the presence of bond paths between the helium atom and the cage carbon atoms. Near that time, Merino et al. [13,14] have studied the effect of symmetry on the bond paths in He@cubane, He@dodecahedrane, He${}_{2}$@dodecahedrane, and Ng@C${}_{60}$ (Ng = He, Ne, Ar, and Kr) and found that the presence of multiple BPs may in fact result from the high symmetry of a system and does not necessarily mean a stabilizing effect. Therefore, making the one-to-one analogy between BP and a chemical bond has proven to be risky [13].

The presence of BP and BCP in such spatially crowded circumstances gave rise to the term “counterintuitive” bond path [15,16,17,18,19,20], which is a bond path that occurs between a pair of atoms for which, based on a variety of data (especially energetic or structural), a stabilizing interaction is not expected. Importantly, such counterintuitive BPs have also been found between many pairs of various atoms, especially strongly electronegative [6,9,15,16,17,21,22,23,24,25,26,27,28,29,30,31,32,33,34] and having a large radius [15,16,23], although the H⋯H interaction is also a good example [6,7,8,35,36,37,38,39]. Many examples of systems containing counterintuitive BP are listed in [15,16,17,18].

In the context of the counterintuitive ‘repulsive’ bond paths, cubane (C${}_{8}$H${}_{8}$), adamantane (C${}_{10}$H${}_{16}$), dodecahedrane (C${}_{20}$H${}_{20}$), and the fullerene C${}_{60}$ have hitherto been taken as the caging host molecules [11,12,13,14]. Of course, demonstrating the presence of a counterintuitive BP is closely related to demonstrating the non-stabilizing (nonbonding) nature of a given interaction, which is related to its energetics. This is where endohedral complexes are also very helpful. For example, when studying endo- and exohedral complexes of many atoms and ions with several cage hydrocarbons, Moran et al. have concluded that “exohedral binding is preferred to endohedral encapsulation without exception” [10].

**Figure 1:**
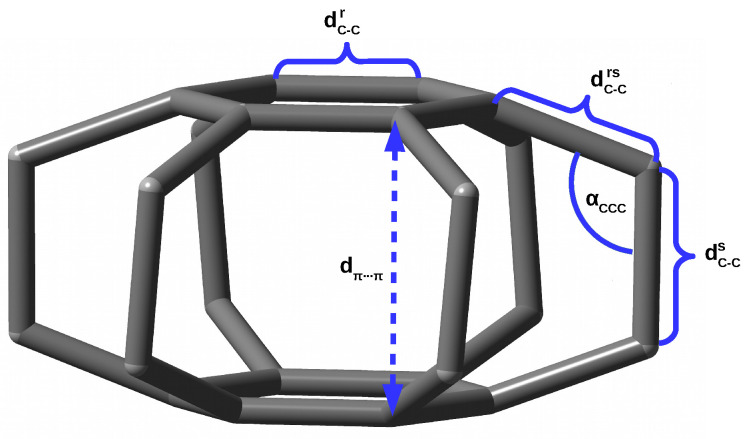
Structure of the superphane (i.e., [2.2.2.2.2.2](1,2,3,4,5,6)cyclophane) molecule (hydrogen atoms are removed for clarity) and labels of the most important geometric parameters.

Some measure of the nature of the interaction between the trapped entity and the host molecule are also the structural changes occurring therein during the formation of the endohedral complex. Unfortunately, these changes are often small or even negligible due to the high stiffness of the guest molecule [40]. I have recently shown [18,19] that the superphane molecule, i.e., [2.2.2.2.2.2](1,2,3,4,5,6)cyclophane [41,42,43,44], is very suitable for studying steric effects in endohedral complexes. This is due to high flexibility of this molecule. Namely, the superphane is made up of two parallel benzene rings linked together by six ethylene bridges (see Figure 1).

These ethylene bridges are structurally flexible enough that the presence of a guesting entity in the interior of the superphane cage is manifested in significant structural changes of the superphane molecule, that are much greater than in all the previously studied systems, e.g., cubane, adamantane, dodecahedrane, C${}_{60}$, etc. For example, inserting a noble gas atom into the cavity of the superphane molecule, i.e., the Ng@superphane endohedral complex formation, leads to its significant “swelling”, which is mainly manifested by increasing the distance between benzene rings ($d_{\pi \cdots \pi }$), lengthening the C-C bridge bonds ($d_{C-C}^{s}$) and increasing the C-C-C angles ($\alpha_{\mathrm{CCC}}$). In the case of Kr@superphane, this effect has turned out to be so large that the linker C-C bond becomes one of the longest (1.753 Å) so far reported [45,46,47,48,49,50,51,52]. The energetic disadvantage of the Ng⋯C interactions in the Ng@superphane complexes has also been confirmed by the positive values of the binding energy. Moreover, their antibonding character has been demonstrated [18] by negative values of Mayer Bond Orders [53,54,55,56].

A certain drawback (in the context of studying the nature of the guest⋯host interaction) of the superphane molecule is that the presence of as many as six ethylene linkers in it prevents or at least significantly hinders the trapped entity from escaping out of the superphane cage. Hence the idea to use superphane derivatives with correspondingly reduced number of ethylene linkers, leading to well-known [2${}_{n}$] cyclophanes [57,58,59,60,61,62], where n<6. Thus, the aim of this article is to show the repulsive effect of the Ng⋯host interaction by using various types of cyclophanes, which should allow the initially trapped Ng atom to escape out of the cyclophane cage much more easily than the superphane molecule possessing as many as six ethylene linkers [18,19]. Such a possible spontaneous, i.e., during geometry optimization, escape of the initially trapped atom out of the cage of a host molecule would be a good evidence that the Ng⋯host interaction inside the Ng@host endohedral complex is, indeed, nonbonding (i.e., repulsive), and not stabilizing (attractive).

## 2. Methodology

Initially, in order to select a reliable exchange-correlation functional [63] of Density Functional Theory (DFT) [64,65], the experimentally (CLOPNA.cif file, deposition number 1127275) [42] and theoretically obtained superphane structures were compared with each other. Thus, several popular functionals such as B3LYP [66,67,68], B3LYP-D3 [66,67,68,69,70], B3PW91 [71], B3PW91-D3 [69,70,71], TPSSh [72,73], M06L [74], M06 [75], M06-HF [76], M06-2X [75], PBE0 [77,78], and ωB97X-D [79] were used. Additionally, the performance of the Hartree–Fock (HF) method [80] was also tested. The 6-311++G(d,p) basis set [81], being of the Valence Triple Zeta (VTZ) type and possessing a set of polarization and diffuse functions on all atoms, was used. Then, geometry optimizations of the considered systems were made at the ωB97X-D/6-311++G(d,p) level of theory. Obtaining minima on the potential energy surfaces was confirmed by the lack of imaginary frequencies in the vibration analysis. Both the geometry optimization and the frequency analysis were performed with the Gaussian 16 program [82].

The deformation (distortion) energy ($E_{\mathrm{def}}$) of the cyclophane molecule is simply the difference between the total energies of the cyclophane with its complex geometry and with its equilibrium geometry, i.e., obtained after its full optimization:

(1) $$E_{\mathrm{def}}=E\left({\mathrm{cyclophane}}^{⋆}\right)-E\left(\mathrm{cyclophane}\right)>0$$

Obviously, the deformation energy is positive, which results from the energetic destabilization due to steric distortions. In order to describe the energetics of the Ng⋯cyclophane interaction, the interaction ($E_{\mathrm{int}}$) and binding $E_{b}$ energies were determined using the following formulas:

(2) $$E_{\mathrm{int}}=E\left(\mathrm{complex}\right)-E_{\mathrm{com}}^{\mathrm{com}}\left(\mathrm{cyclophane}\right)-E^{\mathrm{com}}\left(\mathrm{Ng}\right)$$

(3) $$E_{b}=E\left(\mathrm{complex}\right)-E\left(\mathrm{cyclophane}\right)-E\left(\mathrm{Ng}\right)$$

In the case of $E_{\mathrm{int}}$, the total energies of cyclophane and Ng were calculated in the basis set of the complex, and additionally the cyclophane molecule had the geometry taken from the complex. Thus, $E_{\mathrm{int}}$ takes into account the basis set superposition error (BSSE) [83], although it should be rather small at the DFT level. In contrast, $E_{b}$ requires a fully optimized cyclophane structure and therefore also includes a deformation contribution. Importantly, a negative value of $E_{\mathrm{int}}$ and $E_{b}$ indicates a binding effect of the Ng⋯cyclophane interaction in the complex, while positive values show a non-binding effect.

The Mayer Bond Order (MBO) [53,54,55,56] was computed using the following formula:

(4) $${\mathrm{MBO}}_{\mathrm{AB}}=\sum_{\alpha \in A}\sum_{\beta \in B}{\left(\mathrm{PS}\right)}_{\alpha \beta }{\left(\mathrm{PS}\right)}_{\beta \alpha }$$

where **P** and **S** are the density and atomic orbital overlap matrices, respectively. Negative MBO${}_{\mathrm{AB}}$ value for an A-B bond or interaction indicates the antibonding nature of this bond (interaction).

## 3. Results and Discussion

### 3.1. Experimental vs. Theoretical Structure of the Superphane Molecule

Given the superphane crystallographic structure [42], it is worth testing the performance of various popular exchange-correlation functionals towards the reproducibility of this structure. As mentioned in the Methodology section, such comparative computations were made for B3LYP, B3LYP-D3, B3PW91, B3PW91-D3, TPSSh, M06-L, M06, M06-HF, M06-2X, PBE0, ωB97X-D and, additionally, the Hartree–Fock method. It should be emphasized that despite the widely repeated opinion that superphane has the $D_{6h}$ point group [43,44], in fact its symmetry is ‘only’ $C_{i}$, which results from a tiny twisting of the ethylene linkers, which will be discussed later. Moreover, in the case of computations, the structure with $C_{i}$ symmetry is characterized by the presence of one imaginary frequency. For this reason, reoptimizations were made, which have led to structures lacking symmetry (i.e., featuring $C_{1}$ point group) and therefore Table 1 shows the results for both $C_{i}$ and $C_{1}$.

As for the crystallographic structure of the superphane, let us first note that the dispersion of the values of the formally identical parameters is quite large, and the real point group of the experimentally determined superphane structure [42] is $C_{i}$ (see CLOPNA.cif) and not $D_{6h}$ [43,44]. For example, the values of the unique $d_{\pi \cdots \pi }$ distance pairs are 2.620, 2.623, and 2.630 Å, which give the benzene rings a small fold, and the unique pairs of $d_{C-C}^{s}$ are 1.575, 1.581, and 1.584 Å. These values are averaged to 2.624 and 1.580 Å, respectively, as shown in Figure 1 in reference [43] and Table 1. Importantly, the ethylene bridges in the experimentally described superphane molecule are actually slightly twisted, but the twist angle is very small ($\theta_{\mathrm{CCCC}}$ = 0.2°).

The theoretical results show, however, that the twist angle $\theta_{\mathrm{CCCC}}$ in the $C_{i}$ symmetry superphane is exactly 0°. As for the C-C bond length agreement, it is generally good, with the exception of B3LYP (RMS = 0.015 Å), B3LYP-D3 (0.015 Å), TPSSh (0.014 Å) and M06-HF (0.013 Å). The best agreement is provided by the M06 (0.006 Å), PBE0 (0.007 Å), M06-L (0.008 Å) and ωB97X-D (0.008 Å) functionals. Interestingly, even the Hartree-Fock method is not too bad here (0.010 Å). However, although the $\alpha_{\mathrm{CCC}}$ angle is reproduced with good accuracy (0.0°–0.4°), the Hartree-Fock method gives the least compliance (0.4°). It is worth noting that in the group of methods studied here, B3LYP is the only functional that gives minimum featuring the $C_{i}$ symmetry. Although this corresponds to the crystallographically determined structure [42], this result should be taken as an artifact, because the undoubtedly better (as a result of adding the Grimme dispersion correction [69,70]) B3LYP-D3 functional does not confirm this result, giving $C_{1}$ symmetry.

As mentioned earlier, with the exception of B3LYP, all the other exchange-correlation functionals and the Hartree-Fock method show that a structure with $C_{i}$ symmetry is only a transition state with one imaginary frequency and a minimum lacks symmetry, i.e., belongs to the $C_{1}$ point group. All these methods consistently give a fairly significant twist angle $\theta_{\mathrm{CCCC}}$, from 3.6° in the case of B3LYP-D3 up to ca. 11.5° for M06-L and M06-2X and 12.3° for M06-HF. It should be noted that reoptimizations to the minima with $C_{1}$ symmetry have led to the increased π⋯π distance (see $d_{\pi \cdots \pi }$ in Table 1) by 0.001–0.004 Å depending on the method used. In contrast, the ethylene linker bonds are shortened (except for B3LYP-D3) by 0.001–0.004 Å. There is therefore no doubt that the twisting of the ethylene linkages is intended to reduce the bond strain present in the symmetrical ($C_{i}$) superphane molecule by slightly greater spacing of the benzene rings from each other and shortening the unfavorably elongated C-C linker bonds. It can be assumed that in the superphane crystal lattice [42] this loosening of bond tensions is largely due to interactions with neighboring molecules, as a result of which the twist angle $\theta_{\mathrm{CCCC}}$ is ‘only’ 0.2°. Of course, the effects of the packing of the lattice and the presence of neighboring molecules mean that the compatibility of the crystallographic structure vs. theoretical one must also be treated with some reserve. Finally, we note two trends. First, fully optimized $C_{1}$ structures have a slightly lower RMS value than structures with $C_{i}$ symmetry. Second, it is worth remembering that the B3LYP functional (and its dispersion-corrected version B3LYP-D3) give the worst geometries. Therefore, it is better to use ideologically similar [63] B3PW91 (or B3PW91-D3). However, it is even better to reach for a more appropriate exchange-correlation functional, such as, for example, M06-L, M06, M06-2X, PBE0, and ωB97X-D. Taking into account the obtained results (Table 1) and the fact that ωB97X-D was among the best functionals for general purposes out of 200 tested [63], this functional was selected for the calculations discussed further.

### 3.2. Ng@superphane Complexes

As superphane is treated as a parent molecule, the Ng@superphane (Ng = He, Ne, Ar, Kr) endohedral complexes will be discussed first. Their most important energetic and structural parameters are presented in Table 2.

As already mentioned in the Introduction and in the previous subsection, the superphane molecule (Figure 1) is characterized by the presence of two parallel benzene rings ($d_{C-C}^{r}$ = 1.406 Å) being 2.653 Å apart and linked together by six ethylene bridges being 1.592 Å long. Therefore, these bridges are already significantly longer than the C-C bond in, for example, ethane (1.526 Å at the same level of theory). The $\alpha_{\mathrm{CCC}}$ angles in these bridges are 110.3°. Importantly, the full geometry optimization of the superphane molecule results in a slight twisting of the ethylene bridges, as the $\theta_{\mathrm{CCCC}}$ dihedral angles are 6.7°. These twists prove high angular strains in the molecule.

**Figure 2:**
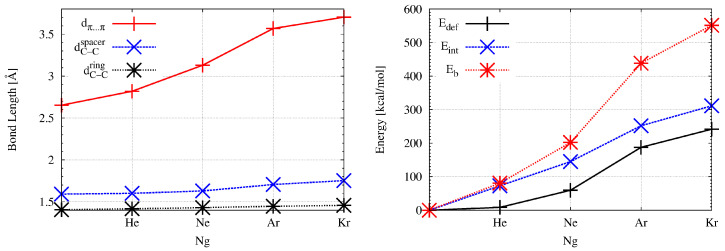
Dependence of the distance $d_{\pi \cdots \pi }$, the length of the bonds $d_{C-C}^{s}$ and $d_{C-C}^{r}$ (**left**) and the interaction, binding, and deformation energy (**right**) on the noble gas atom (Ng = He, Ne, Ar, Kr) in the endohedral Ng@superphane complexes.

Encapsulation of a noble gas atom into the interior of superphane causes significant changes in geometric parameters. These changes clearly indicate the “swelling” of the superphane molecule, which increases with increasing radius of the Ng atom; He→Ne→Ar→Kr. This is also shown in Figure 2 to better illustrate the scale of the changes.

As can clearly be seen, the biggest changes concern the distance between the rings ($d_{\pi \cdots \pi }$). Already inserting a helium atom increases this distance by more than 6%, from the initial value of 2.653 Å to 2.819 Å. Converting He to Ne gives a further increase of 11% (to 3.130 Å), which is 18% of the value in superphane. However, the greatest percentage increase (14%) occurs upon the Ne→Ar replacement and the π⋯π distance is 3.568 Å. This is a 0.915 Å increment (ca. 34.5%) over the superphane value. The Ar→Kr replacement is not so spectacular, although the distance of 3.703 Å is almost 40% longer than in the superphane molecule. Such a large increase in the distance between benzene rings clearly shows both the large trapping potential of the superphane molecule as well as its high structural flexibility [18]. Importantly, subsequent changes He→Ne→Ar→Kr also lead to significant elongations of the already long C-C bonds in ethylene bridges. In the case of the Kr@superphane complex, this length is as high as 1.753 Å (thus 0.161 Å, i.e., 10%, longer than in superphane), which pushes these bonds to some of the longest ever reported [45,46,47,48,49,50,51,52]. Although such a large extension of the C-C bonds did not cause the Kr atom to escape from the cage of the superphane molecule, such an event is possible at the level of some of the weaker computational protocols [18]. In addition to the aforementioned increases in the values of $d_{\pi \cdots \pi }$ and $d_{C-C}^{s}$, it is also worth noting a clear expansion of the size of both benzene rings, whereby the encpsulation of the Kr atom leads to two values of the C-C bond lengths in these rings, which occur alternately. The increase in the size of the superphane, i.e., its “swelling”, caused by the insertion of a noble gas atom inside it, is also clearly visible in the increase in $\alpha_{\mathrm{CCC}}$ (Table 2). In the case of Kr@superphane, this angle is as much as ca. 128°compared to ca. 110° in the superphane. Thus, the spacing of the benzene rings from one another entails a visible ‘opening’ of the C-C-C angles and hence a certain straightening of the ethylene bridges. Encaspulation also has some influence on the amount of bending of the ethylene bridges in the complex, namely it increases slightly in the case of He, Ar and especially Ne (9.7°), while in the case of the largest Kr, the $\theta_{\mathrm{CCCC}}$ angle decreases slightly and differentiates (3.2° and 5.0°).

It is obvious that such large structural distortions in the superphane molecule have an impact on its energetic instability, which can be measured by means of the deformation energy ($E_{\mathrm{def}}$ in Table 2) defined by Equation (1). The encapsulation of the He atom inside the superphane molecule gives the value of 8.5 kcal/mol, but $E_{\mathrm{def}}$ quickly increases with the radius of the Ng atom (Figure 2) and in the Kr@superphane complex it reaches a value of as much as 241.7 kcal/mol. It is worth mentioning that $E_{\mathrm{def}}$ apparently depends on the size of the cavity in the host molecule and for example in the smaller He@adamantane endohedral complex it amounts to 15.3 kcal/mol [11,12].

The fundamental issue in this discussion is the energetic of the Ng⋯host interaction. As mentioned in the Methodology section, for this purpose the interaction $E_{\mathrm{int}}$ and binding $E_{b}$ energies were calculated. The most important result is that both of these energies, i.e., $E_{\mathrm{int}}$ and $E_{b}$ are positive, and therefore the Ng⋯superphane interactions inside the Ng@superphane complexes are non-bonding [18]. Obviously, the magnitude of the non-bonding effect increases significantly with the size of the Ng atom (Figure 2) and reaches the highest value in the Kr@superphane complex ($E_{\mathrm{int}}$ and $E_{b}$ amount to 311.3 kcal/mol and 551.5 kcal/mol, respectively). It is obvious, therefore, that both the strong structural deformations of the superphane molecule along with high values of the deformation energy as well as large and positive values of the interaction and binding energies show that the encapsulation of a noble gas atom inside the superphane molecule is an energetically unfavorable effect.

In order to even better illustrate the repulsive nature of the Ng⋯host interaction inside the cage of the host molecule, some modifications can be made to its structure to make it easier for the trapped atom to escape from the inside of the cage. This is the idea behind this article. A fairly natural idea, but not the only one, is to gradually remove the ethylene bridges, leading to cyclophanes [2${}_{n}$] with n<6 (recall that superphane is [2.2.2.2.2.2](1,2,3,4,5,6)cyclophane that is [2${}_{6}$](1,2,3,4,5,6)cyclophane, for which *n* = 6) [41,42,43,44]. As already mentioned in the Introduction, [2${}_{n}$] cyclophane is a rich family of compounds of great interest [57,58,59,60,61,62]. The discussion of what happens after the removal of at least one of the ethylene bridges of the superphane molecule is carried out in the next subsection.

### 3.3. Opening the Cage

**Figure 3:**
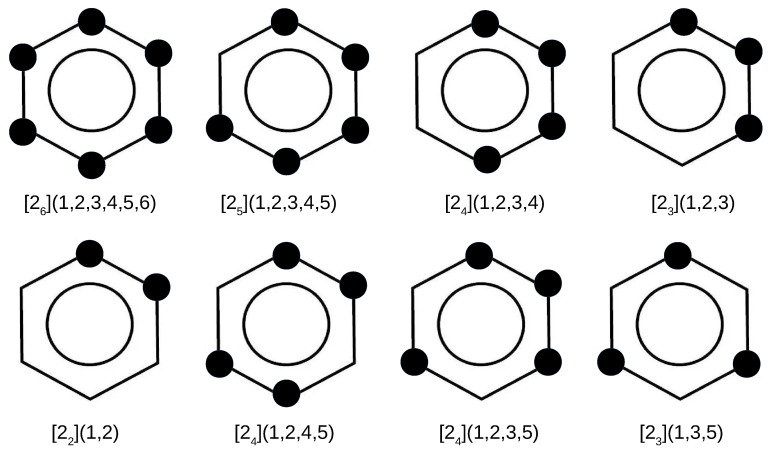
Considered cyclophanes. Large dots indicate the presence of ethylene bridges linking two benzene rings of the cyclophane molecule.

In addition to the superphane molecule, which, along with its endohedral complexes Ng@superphane, was discussed in the previous subsection, an attempt to encapsulate Ng into the interior of another 7 cyclophanes has been investigated. Together with the parent superphane, they are schematically depicted in Figure 3, where large dots indicate the presence of ethylene linkers between the benzene rings of the corresponding cyclophane molecule.

**Figure 4:**
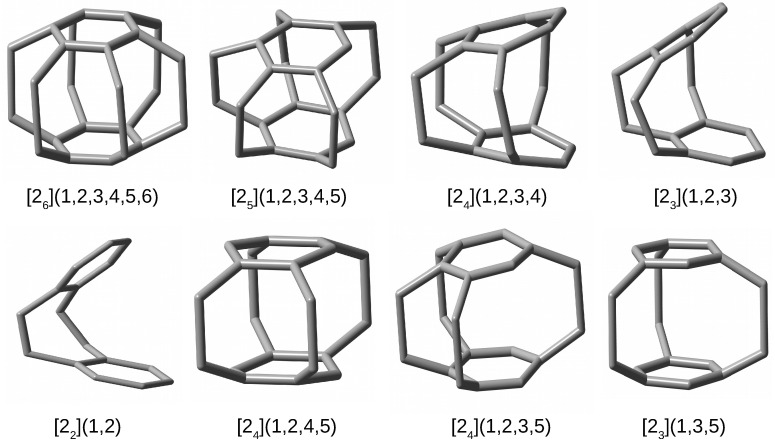
Structures of cyclophanes (hydrogen atoms have been removed for better visualization of the carbon backbones).

Additionally, to facilitate the spatial visualization of the discussed cyclophanes, their structures are shown in Figure 4, with all hydrogen atoms removed to better visualize the carbon skeletons.

By comparing the structures of the first five cyclophanes, i.e., [2${}_{6}$](1,2,3,4,5,6) (superphane), [2${}_{5}$](1,2,3,4,5), [2${}_{4}$](1,2,3,4), [2${}_{3}$](1,2,3) and [2${}_{2}$](1,2), immediately noticeable is the increasing inclination of the benzene rings in relation to each other, so that these molecules are more and more open. In particular, the cyclophanes [2${}_{3}$](1,2,3) and [2${}_{2}$](1,2) resemble open shells, so that talking about cages in these cases is a kind of abuse. Nevertheless, they were also considered to better support the conclusions obtained. The other cyclophanes, on the other hand, do create a cage in their interior. It is worth noting that in the context of the conducted research, the most interesting systems are [2${}_{5}$](1,2,3,4,5)cyclophane and then [2${}_{4}$](1,2,4,5)cyclophane, [2${}_{4}$](1,2,3,5)cyclophane, and [2${}_{3}$](1,3,5)cyclophane, because they are characterized by the presence of only single-carbon windows (Figure 3). Thus, any escape of the initially trapped Ng atom is allowed, but only to the smallest extent.

The complexes of the Ng atom with [2${}_{5}$](1,2,3,4,5)cyclophane, [2${}_{4}$](1,2,3,4)cyclophane, [2${}_{3}$](1,2,3)cyclophane, and [2${}_{2}$](1,2)cyclophane will be discussed first, as such a set allows for the analysis of the influence of the gradual enlargement of the window size in the cyclophane molecule. The parameters allowing for the structural and energetic characteristics of these complexes are shown in Table 3. Due to the possible differentiation of the values of certain geometric parameters, in these cases they are given in the format $v_{\min}$–$v_{\max}$, where $v_{\min}$ and $v_{\max}$ denote the minimum and maximum values, respectively. However, the trends of their obtained changes are more important than the values themselves.

**Figure 5:**
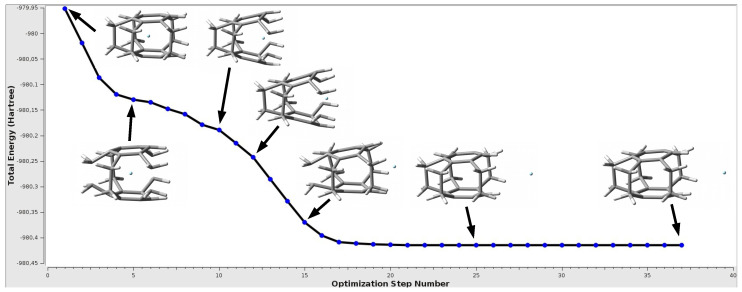
The change in the total energy during the geometry optimization of the Ne@[2${}_{5}$](1,2,3,4,5)cyclophane endohedral complex.

The most important result of this part of the research is the finding that only in the first complex from Table 3, i.e., for [2${}_{5}$](1,2,3,4,5)cyclophane and He, the trapped He atom remains inside the cyclophane cage with formation of the He@[2${}_{5}$](1,2,3,4,5)cyclophane endohedral complex. In all the other cases, however, the initially trapped Ng atom escapes from the interior of the cyclophane molecule, thus creating a cyclophane⋯Ng exohedral complex. This is another strong proof that the interaction between the trapped Ng atom and the interior of the cyclophane is highly destabilizing, i.e., repulsive. If it were not so, one may ask why the trapped Ng atom so willingly leaves the interior of the cyclophane. There is no question of a possibly higher energetic local minimum corresponding to the endohedral complex, because such a minimum does not actually exist and the ejection of the Ng atom from the interior of the cyclophane occurs spontaneously during the geometry optimization of the initially built endohedral complex. One such representative case is shown in Figure 5.

As can be seen, the initially trapped Ne atom approaches the window of the [2${}_{5}$](1,2,3,4,5) cyclophane molecule, which causes its significant increase and breaks the parallelism of benzene rings, and then Ne leaves the cyclophane molecule, creating a minimum on a flat fragment of the potential energy surface, corresponding to the [2${}_{5}$](1,2,3,4,5)cyclophane⋯Ne exohedral complex. The whole process is associated with a monotonic decrease of the total energy of the complex. Of course, since the entity that interacts with the cyclophane is a noble gas atom, the interaction energy in the exodohedral complex is very small, equal to only −0.5 kcal/mol ($E_{b}$ = −1.0 kcal/mol). It is noteworthy that after the formation of the exohedral complex with Ne, the [2${}_{5}$](1,2,3,4,5)cyclophane molecule is closed again as it was in the initially modeled endohedral complex, and the changes in geometric parameters are negligible (compare lines 1 and 3 in Table 3) so that the deformation energy of the cyclophane molecule is zero. An almost identical situation also occurs in the case of Ar and Kr and the [2${}_{4}$](1,2,3,4)cyclophane⋯Ng complexes, although in the latter case there are slightly larger changes in the distance π⋯π (actually C${}_{\mathrm{ring}}\cdots$C${}_{\mathrm{ring}}$), which results from a slightly more pronounced boat conformation of the rings.

**Figure 6:**
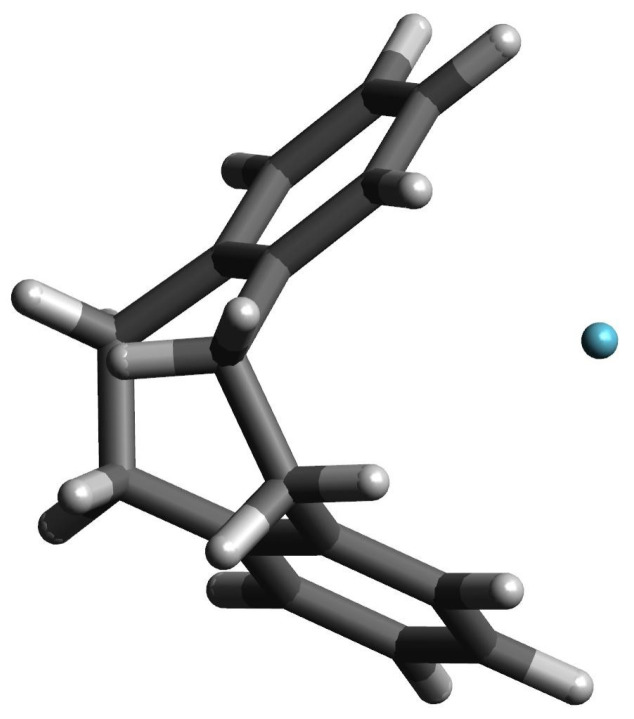
The structure of the [2${}_{2}$](1,2)cyclophane⋯Kr complex.

However, the greatest differences in π⋯π (i.e., C${}_{\mathrm{ring}}\cdots$C${}_{\mathrm{ring}}$) occur in the case of Ng complexes with either [2${}_{3}$](1,2,3)cyclophane or [2${}_{2}$](1,2)cyclophane, which results from the fact that these molecules do not completely return to their original, i.e., before the complex geometry optimization, structures. Especially for the latter molecule (and for the largest Ar and Kr), clearly greater opening of the cyclophane structure and slightly greater twisting of the ethylene linkages are visible. Still however, the deformation energy is practically zero and only in the [2${}_{2}$](1,2)cyclophane⋯Kr complex does it amount to 0.3 kcal/mol. This result shows that even a significant increase in the distance between the benzene rings and a slightly greater twisting of the ethylene bridges in [2${}_{2}$](1,2)cyclophane do not require a large energy input. This is most likely due to the relatively large distance between these rings (see Figure 4) and therefore the relatively weak interaction between them. The structure of [2${}_{2}$](1,2)cyclophane⋯Kr complex is shown in Figure 6 to illustrate an exemplary location of the Ng atom.

**Figure 7:**
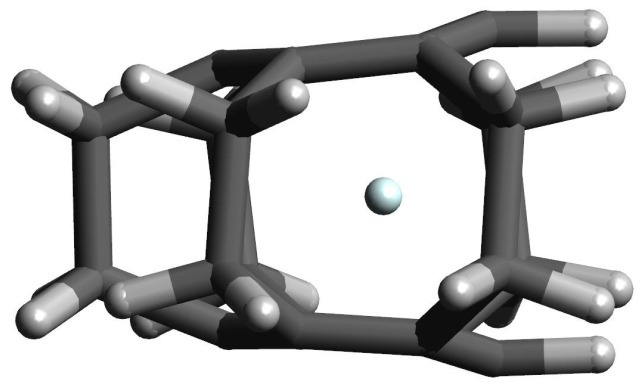
The structure of the He@[2${}_{5}$](1,2,3,4,5)cyclophane complex.

Let us return, however, to the interesting example of the He@[2${}_{5}$](1,2,3,4,5)cyclophane endohedral complex, in which the trapped He atom remains inside the [2${}_{5}$](1,2,3,4,5)cyclo- phane molecule. Of course, as in the case of the Ng@superphane (i.e., Ng@[2${}_{6}$](1,2,3,4,5,6)cy- clophane) complexes, both the interaction energy and the binding energy are positive (59.0 kcal/mol and 69.7 kcal/mol, respectively; Table 3). It is worth noting, however, that both values are 13.8 kcal/mol and 11.0 kcal/mol lower than for He@superphane (Table 2). This is due to the fact that the trapped He atom remains inside the [2${}_{5}$](1,2,3,4,5)cyclophane molecule, but shifts slightly towards its window (see Figure 7), thus slightly lowering the unfavorable internal cage repulsion. Certainly the slightly boat-shaped conformation of the rings is also of some importance here (see Figure 4), with the result that the carbon atoms on the window side of the [2${}_{5}$](1,2,3,4,5)cyclophane molecule are slightly further away (3.012 Å) than the carbon atoms in the *meta* (2.628 Å) or *para* (2.651 Å) positions. The comparison of the first two lines in Table 3 clearly shows that also in this case the encaspuslation leads to a significant ‘swelling’ of the cyclophane molecule, which is manifested by an increase in the distance between benzene rings, expansion of their size, elongations of the C-C linker bonds and greater ‘opening’ of the C-C-C angles. Moreover, the greater twisting of the ethylene linkers is also visible.

The other cyclophanes considered, i.e., [2${}_{4}$](1,2,4,5), [2${}_{4}$](1,2,3,5) and [2${}_{3}$](1,3,5), are of particular interest mainly for two reasons, namely quite high symmetry (D${}_{2h}$, C${}_{2v}$ and D${}_{3h}$, respectively) and, as already mentioned, having only single-carbon windows (see Figure 3). Thanks to these features, these cyclophanes have a really closed structure, creating spatial cages inside them. Moreover, the presence of only single-carbon windows allows for an escape of the trapped atom, but this escape should be much more difficult than in the case of cyclophanes [2${}_{4}$](1,2,3,4), [2${}_{3}$](1,2,3), or [2${}_{2}$](1,2). The energetic and geometrical parameters for cyclophanes [2${}_{4}$](1,2,4,5), [2${}_{4}$](1,2,3,5), and [2${}_{3}$](1,3,5) and their complexes with Ng are presented in Table 4.

**Figure 8:**
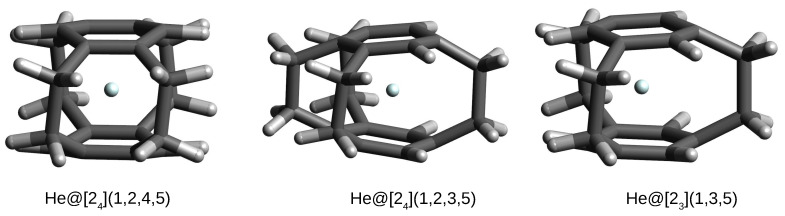
Structures of some He@cyclophane complexes.

The most important result is that only in the case of systems with the He atom, this atom remains in the interior of the considered cyclophanes, forming endohedral complexes. However, in the case of the remaining Ng, i.e., Ne, Ar, and Kr, these atoms escape from the cyclophane cages to form exohedral complexes. This result again indicates the highly repulsive effect of the cyclophane cages on the entity placed inside them. The three cases of helium endohedral complexes are shown in Figure 8.

Of course, as could be expected, these complexes are characterized by positive interaction and binding energies, thus proving the non-bonding nature of He⋯cyclophane interactions inside the cages of the respective cyclophanes. The interaction energies are similar to each other and slightly lower (ca. 54–56 kcal/mol) than that obtained for He@[2${}_{5}$](1,2,3,4,5) (59.0 kcal/mol; Table 3). This is most likely the result of lower strain due to the presence of only 3–4, not 5, ethylene bridges (Figure 3). A similar situation applies to the binding energy, ca. 64–66 kcal/mol vs. 70 kcal/mol in He@[2${}_{5}$](1,2,3,4,5). The He atom trapping in the case of these three cyclophanes is also slightly less energetically unfavorable for the cyclophane molecules than for [2${}_{5}$](1,2,3,4,5)cyclophane in the He@[2${}_{5}$](1,2,3,4,5)cyclophane complex ($E_{\mathrm{def}}$ amounts to ca. 9–10 kcal/mol vs. 11 kcal/mol, respectively). It is similarly visible (Table 4) that the encapsulation of the He atom inside these three cyclophanes leads to their ‘swelling’. For example, in the case of the He@[2${}_{3}$](1,3,5)cyclophane complex, the $d_{\pi \cdots \pi }$ distances increase from 2.773–2.858 Å in the [2${}_{3}$](1,3,5)cyclophane to 2.990–3.124 Å in the complex, and the C-C spacer and ring bonds elongate from 1.611 Å and 1.394 Å, respectively, to 1.628 Å and 1.402 Å, respectively. Moreover, the C-C-C- angle also opens up more, from 112.6° to 116.5°. It is worth noting that the encapsulation of the He atom inside these cyclophanes leads to a significant (up to 11°) twisting of the initially straight ethylene bridges.

As already mentioned, in all the other cases shown in Table 4, the initially trapped Ng atom escapes from the cyclophane cage with formation of an exohedral complex. However, there are two possible variants that should be highlighted. In one of these variants, the initially trapped Ng atom exits the cyclophane cage to form an exohedral complex in which the cyclophane molecule is reconstructed (as in Figure 5) and therefore the deformation energy is zero. This is the case of the Ne complexes and one of the two found forms of the [2${}_{3}$](1,3,5)cyclophane⋯Ar complex.

**Figure 9:**
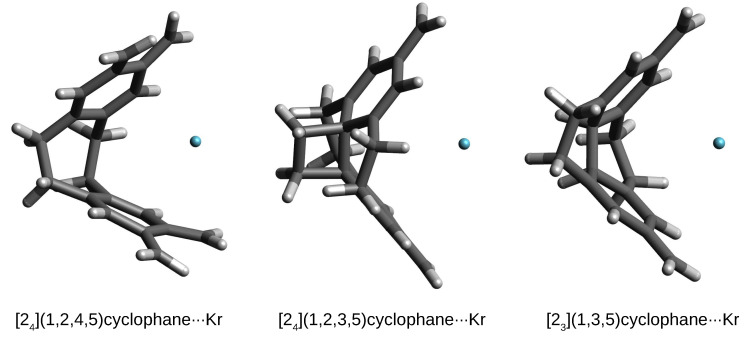
Structures of some cyclophane⋯Kr complexes.

From the point of view of structural changes, the second variant, concerning the larger Ar and Kr atoms, is much more interesting. Namely, in these cases, the initially trapped either Ar or Kr atom is also thrown out of the cyclophane cage, but at a shorter distance, and, most importantly, the structure of the cyclophane molecule is practically destroyed, which is associated with high deformation energy values (Table 4). This is shown in Figure 9 on the example of the corresponding Kr complexes.

As can be clearly seen, the cyclophane molecules accept a shell-like open form. Consequently, the values of $E_{\mathrm{def}}$ are quite significant, especially in the case of [2${}_{4}$](1,2,4,5)cyclophane⋯Kr (53.7 kcal/mol). Although the formation of such complexes is energetically disadvantageous, the interaction energy is quite high (ca. –3 kcal/mol), comparable, for example, to the interaction energies for weak hydrogen bonds [84,85,86,87,88]. With such a large destruction of the original structure of the cyclophane molecule, new interesting structural forms may arise. For example, in the case of the [2${}_{4}$](1,2,3,5)cyclophane⋯Kr complex, the C-C-C angles in one of the ethylene bridges decrease to 91°, which makes it possible to form an almost square carbon ring (well visible in Figure 9). The opening of the cyclophane molecules associated with the breaking of some ethylene bridges allows for the reduction of bond tension by significant twisting of the remaining ethylene bridges, which in the Kr (or Ar) complexes with [2${}_{4}$](1,2,4,5)cyclophane or [2${}_{3}$](1,2,3)cyclophane takes the value of ca. 30°.

### 3.4. Mayer Bond Order

As mentioned in the Introduction, MBO has been used in my recent studies of the Ng@superphane endohedral complexes [18]. The obtained negative values for the Ng⋯π (in fact Ng⋯C${}_{\mathrm{ring}}$) interaction showed that these interactions in these complexes are antibonding, which confirmed my conclusion regarding their destabilizing nature. As has been shown, by performing further studies on Ng complexes with various cyclophanes, yet another four endohedral complexes (He@[2${}_{5}$](1,2,3,4,5)cyclophane, He@[2${}_{4}$](1,2,4,5)cyclophane, He@[2${}_{4}$](1,2,3,5)cyclophane, and He@[2${}_{3}$](1,3,5)cyclophane) have been found. Therefore, it is interesting to determine the MBO values for the He⋯C${}_{\mathrm{ring}}$ interactions also for these complexes. Due to the fact that, unlike in superphane, the benzene rings in the remaining cyclophanes are somewhat folded (see Figure 4), the MBO${}_{\mathrm{He}\cdots C}$ values were determined for each of the ring carbon atoms. *Negative* values have been obtained for each of these He⋯C${}_{\mathrm{ring}}$ contacts and for each of these endohedral complexes, indicating their antibonding nature. Averaged values are similar to each other and are as follows: −0.030 for He@[2${}_{5}$](1,2,3,4,5)cyclophane, −0.031 for He@[2${}_{4}$](1,2,4,5)cyclophane, and He@[2${}_{4}$](1,2,3,5)cyclophane, −0.033 for He@[2${}_{3}$](1,3,5)cyclophane. This is another argument that the Ng⋯π interactions in endohedral complexes are destabilizing [18]. It is worth adding here that negative MBO values have also been found more recently for some cation⋯C interactions in endohedral cation@superphane complexes [19] and for X⋯π (X = H, F, Cl) interactions in *in* forms of some “iron maiden” systems [20].

## 4. Conclusions

Although, as I have shown recently [18,19], the superphane molecule, i.e., [2${}_{6}$](1,2,3,4,5,6) cyclophane, is excellent at studying the nature of the guest⋯host interactions in endohedral complexes, its fully closed structure, due to the presence of up to six ethylene bridges linking the two benzene rings, makes it practically impossible for the trapped entity to escape from the superphane cage. This escape, however, is considerably facilitated in superphane derivatives with n<6, i.e., a reduced number of ethylene bridges, which leads to the presence of at least one single-carbon window. By forming 28 (not including the 4 complexes of the parent superphane molecule) Ng@cyclophane endohedral complexes (Ng = He, Ne, Ar, Kr; cyclophane = [2${}_{5}$](1,2,3,4,5)cyclophane, [2${}_{4}$](1,2,3,4)cyclophane, [2${}_{3}$](1,2,3)cyclophane, [2${}_{2}$](1,2)cyclophane, [2${}_{4}$](1,2,4,5)cyclophane, [2${}_{4}$](1,2,3,5)cyclophane, and [2${}_{3}$](1,3,5)cyclophane), it has been shown that in the vast majority of cases the initially trapped Ng atom spontaneously escapes from the cyclophane cage, forming an exohedral complex. This is further [18] evidence showing that the Ng⋯host interaction in the host cage is indeed repulsive, i.e., destabilizing.

From the point of view of the structural changes taking place in the cyclophane molecule, two types of the cyclophane⋯Ng exohedral complexes can be formed. Namely, after the formation of the exohedral complex, the cyclophane molecule can either be completely rebuilt or almost completely destroyed. Obviously, the former case is characterized by zero value of the deformation energy for the cyclophane molecule and its negligible structural changes, whereas in the latter case the deformation energy is significant, as well as the structural changes being visible, e.g., as an opening of the structure resulting from a significant inclination of the benzene rings of cyclophane.

Apart from the parent ‘sealed’ superphane molecule, endohedral complexes are formed only in the case of the smallest He atom trapped by the cyclophanes, featuring only single-carbon windows. Otherwise, the He atom also escapes from inside the cyclophane. However, it has been shown that even in these endohedral complexes the He⋯cyclophane interaction inside the cyclophane cage is nonbonding, as indicated by positive values of interaction and binding energies. This conclusion has been supported by negative values of Mayer Bond Orders, indicating the antibonding nature of He⋯C${}_{\mathrm{ring}}$ interactions inside the cyclophane cages. This highly unfavorable energetically effect causes ‘swelling’ of the cyclophane molecule, which is manifested by increasing the distance between benzene rings and their expansion, significant elongation of the C-C ethylene linker bonds, and an increase in C-C-C angles. Moreover, encapsulation leads to twisting of the ethylene bridges.

The results of the studies presented here can be confronted with the numerous bond paths that appear between the guest atoms and the host atoms (e.g., Ng⋯C) in diverse endohedral complexes [13,14]. It is therefore clear that these bond paths should be regarded as counterintuitive [15,16,17,18,19,20] and that their presence is not at all, as many still believe, evidence of interatomic stabilization.

## Figures and Tables

**Table 1:** The mean values (in Å or degrees) of the most important geometric parameters (see Figure 1) and the RMS for the bonds calculated for the superphane molecule in the $C_{i}$ and $C_{1}$ symmetry.

Method	Symm.	$d_{\pi \cdots \pi }$	$d_{C-C}^{r}$	$d_{C-C}^{s}$	$d_{C-C}^{\mathrm{rs}}$	$\alpha_{\mathrm{CCC}}$	$\theta_{\mathrm{CCCC}}$	RMS (Bonds)
exp. [42]	$C_{i}$ ^*a*^	2.624 ^*b*^	1.406 ^*c*^	1.580 ^*d*^	1.518 ^*e*^	110.1 ^*f*^	0.2 ^*g*^	n/a
HF	$C_{i}$	2.662	1.401	1.594	1.525	110.5	0.0	0.010
	$C_{1}$	2.663	1.401	1.593	1.526	110.4	6.1	0.009
B3LYP	$C_{i}$	2.661	1.412	1.605	1.522	110.3	0.0	0.015
B3LYP-D3	$C_{i}$	2.663	1.412	1.605	1.522	110.3	0.0	0.015
	$C_{1}$	2.665	1.412	1.605	1.552	110.4	3.6	0.015
B3PW91	$C_{i}$	2.639	1.409	1.595	1.515	110.1	0.0	0.009
	$C_{1}$	2.641	1.409	1.594	1.516	110.1	5.3	0.009
B3PW91-D3	$C_{i}$	2.642	1.409	1.595	1.515	110.2	0.0	0.009
	$C_{1}$	2.645	1.409	1.594	1.516	110.2	6.8	0.008
TPSSh	$C_{i}$	2.640	1.412	1.604	1.519	109.9	0.0	0.014
	$C_{1}$	2.643	1.412	1.602	1.520	109.9	8.1	0.013
M06-L	$C_{i}$	2.646	1.409	1.591	1.513	110.4	0.0	0.008
	$C_{1}$	2.650	1.409	1.587	1.515	110.2	11.4	0.005
M06	$C_{i}$	2.641	1.406	1.589	1.512	110.4	0.0	0.006
	$C_{1}$	2.642	1.406	1.587	1.513	110.3	5.7	0.005
M06-HF	$C_{i}$	2.642	1.404	1.602	1.523	109.9	0.0	0.013
	$C_{1}$	2.645	1.404	1.598	1.525	109.7	12.3	0.011
M06-2X	$C_{i}$	2.653	1.408	1.596	1.520	110.4	0.0	0.009
	$C_{1}$	2.657	1.407	1.592	1.521	110.2	11.5	0.007
PBE0	$C_{i}$	2.632	1.407	1.591	1.513	110.1	0.0	0.007
	$C_{1}$	2.635	1.407	1.589	1.513	110.1	7.5	0.006
ωB97X-D	$C_{i}$	2.651	1.406	1.594	1.519	110.4	0.0	0.008
	$C_{1}$	2.653	1.406	1.592	1.519	110.3	6.7	0.007

^*a*^ While there is widespread information that superphane has a $D_{6h}$ point group [43,44], it actually only shows $C_{i}$. ^*b*^ The unique value pairs are 2.620, 2.623, and 2.630 Å. As a consequence, the benzene rings are somewhat folded. ^*c*^ The values are 1.404, 1.405, 1.405, 1.406, 1.408, and 1.408 Å. ^*d*^ The unique value pairs are 1.575, 1.581, and 1.584 Å. ^*e*^ The values are 1.514, 1.517, 1.518, 1.519, 1.519, and 1.522 Å. ^*f*^ The values are 109.9°, 109.9°, 110.1°, 110.2°, 110.3°, and 110.4°. ^*g*^ The value pairs are 0.1°, 0.3°, and 0.3° (of course, opposite dihedrals differ in sign).

**Table 2:** Interaction, binding, and deformation energies (in kcal/mol) and selected geometric parameters (in Å or degrees) for superphane (∅) and the Ng@superphane (Ng = He, Ne, Ar, Kr) complexes.

Ng	$E_{\mathrm{int}}$	$E_{b}$	$E_{\mathrm{def}}$	$E_{\mathrm{def}}^{\%}$	$d_{\pi \cdots \pi }$	$d_{C-C}^{s}$	$d_{C-C}^{r}$	$\alpha_{\mathrm{CCC}}$	$\theta_{\mathrm{CCCC}}$
∅	n/a	n/a	n/a	n/a	2.653	1.592	1.406	110.3	6.7
He	72.8	80.7	8.5	10.5	2.819	1.601	1.417	113.3	8.1
Ne	145.1	202.1	59.5	29.4	3.130	1.630	1.431	118.8	9.7
Ar	251.9	438.4	187.6	42.8	3.568	1.707	1.447	126.1	8.8
Kr	311.3	551.5	241.7	43.8	3.703	1.753	1.434 ${}^{a}$	127.9	3.2 ${}^{a}$
							1.481 ${}^{a}$		5.0 ${}^{a}$

^*a*^ A pair of significantly different values that alternate have been found.

**Table 3:** Interaction, binding, and deformation energies (in kcal/mol), and selected geometric parameters (in Å or degrees) for the cyclophane molecule (if ∅ mark is used) and either the Ng@cyclophane (if Ng is marked with boldface) or cyclophane⋯Ng (Ng = He, Ne, Ar, Kr) complexes obtained after geometry optimizations of the initially built Ng@cyclophane complexes.

Cyclophane	Ng	$E_{\mathrm{int}}$	$E_{b}$	$E_{\mathrm{def}}$	$d_{\pi \cdots \pi }$ ^*a*^	$d_{C-C}^{s}$ ^*a*^	$d_{C-C}^{r}$ ^*a*^	$\alpha_{\mathrm{CCC}}$ ^*a*^	$\theta_{\mathrm{CCCC}}$ ^*a*^
[2${}_{5}$](1,2,3,4,5)	∅	n/a	n/a	n/a	2.628–3.012	1.582–1.603	1.391–1.409	110.0–112.1	5.9–6.1
	**He**	59.0	69.7	11.0	2.758–3.458	1.584–1.627	1.402–1.414	112.3–116.7	8.6–9.6
	Ne	−0.5	−1.0	0.0	2.628–3.014	1.582–1.603	1.391–1.409	110.0–112.2	5.9–6.2
	Ar	−1.1	−1.3	0.0	2.628–3.015	1.582–1.603	1.391–1.409	110.0–112.2	5.7–5.8
	Kr	−1.7	−1.9	0.0	2.628–3.017	1.582–1.603	1.391–1.410	110.0–112.2	5.5–5.6
[2${}_{4}$](1,2,3,4)	∅	n/a	n/a	n/a	2.617–3.479	1.577–1.594	1.384–1.410	109.7–113.4	9.6–11.1
	He	−0.2	−0.2	0.0	2.617–3.478	1.577–1.594	1.384–1.410	109.7–113.4	9.5–11.1
	Ne	−0.5	−0.9	0.0	2.617–3.478	1.577–1.594	1.384–1.410	109.7–113.4	9.8–11.3
	Ar	−1.1	−1.2	0.0	2.617–3.483	1.577–1.594	1.384–1.410	109.7–113.4	9.7–11.3
	Kr	−1.6	−1.8	0.0	2.617–3.486	1.577–1.594	1.384–1.410	109.7–113.4	9.6–11.3
[2${}_{3}$](1,2,3)	∅	n/a	n/a	n/a	2.595–4.470	1.555–1.585	1.388–1.404	109.6–114.9	14.6–18.0
	He	−0.2	−0.2	0.0	2.594–4.464	1.555–1.585	1.388–1.404	109.6–114.8	14.4–17.8
	Ne	−0.5	−0.9	0.0	2.594–4.469	1.555–1.585	1.388–1.404	109.6–114.9	14.4–17.9
	Ar	−1.0	−1.2	0.0	2.594–4.481	1.555–1.585	1.388–1.404	109.6–114.9	14.3–17.9
	Kr	−1.6	−1.8	0.0	2.594–4.496	1.555–1.585	1.388–1.404	109.7–114.9	14.4–18.0
[2${}_{2}$](1,2)	∅	n/a	n/a	n/a	2.926–5.586	1.553	1.388–1.403	113.1–116.9	31.4
	He	−0.2	−0.2	0.0	2.933–5.627	1.553	1.388–1.403	113.2–117.1	31.8
	Ne	−0.5	−1.0	0.0	2.943–5.689	1.553	1.387–1.403	113.4–117.2	32.2
	Ar	−1.4	−1.5	0.1	2.965–5.821	1.551	1.387–1.403	113.7–117.6	33.5
	Kr	−2.3	−2.3	0.3	2.987–5.941	1.550	1.387–1.403	114.0–117.9	34.8

^*a*^ Due to the diversity of values occurring in some cases, the minimum and maximum values are shown in the $v_{\min}$–$v_{\max}$ format.

**Table 4:** Interaction, binding, and deformation energies (in kcal/mol), and selected geometric parameters (in Å or degrees) for the cyclophane molecule (if ∅ mark is used) and either the Ng@cyclophane (if Ng is marked with boldface) or cyclophane⋯Ng (Ng = He, Ne, Ar, Kr) complexes obtained after geometry optimizations of the initially built Ng@cyclophane complexes.

Cyclophane	Ng	$E_{\mathrm{int}}$	$E_{b}$	$E_{\mathrm{def}}$	$d_{\pi \cdots \pi }$ ^*a*^	$d_{C-C}^{s}$ ^*a*^	$d_{C-C}^{r}$ ^*a*^	$\alpha_{\mathrm{CCC}}$ ^*a*^	$\theta_{\mathrm{CCCC}}$ ^*a*^
[2${}_{4}$](1,2,4,5)	∅	n/a	n/a	n/a	2.713–2.982	1.594	1.394–1.400	111.7	0.0
	**He**	56.0	65.0	9.4	2.916–3.267	1.604	1.402–1.408	115.2–115,4	11.2
	Ne	−0.5	−0.9	0.0	2.713–2.984	1.594	1.394–1.400	111.7	0.0
	Ar	−1.9	51.4	53.6	2.927–5.644	1.554	1.344–1.485	113.1–117.5	29.6
	Kr	−3.0	50.3	53.7	2.940–5.722	1.553	1.344–1.485	113.3–117.8	30.2
[2${}_{4}$](1,2,3,5)	∅	n/a	n/a	n/a	2.612–2.917	1.577–1.610	1.391–1.405	110.0–113.0	0.0
	**He**	55.8	65.7	10.3	2.738–3.241	1.577–1.641	1.401–1.412	112.3–118.0	8.9–10.8
	Ne	−0.5	−1.0	0.0	2.612–2.916	1.578–1.612	1.391–1.405	110.0–113.0	0.3
	Ar	−2.0	26.6	28.9	1.609–6.036	1.540–1.546	1.336–1.504	90.9–113.9	9.6–26.1
	Kr	−3.2	25.4	28.9	1.609–6.058	1.540–1.545	1.336–1.504	90.9–113.9	9.6–26.4
[2${}_{3}$](1,3,5)	∅	n/a	n/a	n/a	2.773–2.858	1.611	1.394	112.6	0.0
	**He**	54.2	63.5	9.7	2.990–3.124	1.628	1.402	116.5	9.0
	Ne	−0.5	−0.9	0.0	2.772–2.857	1.613	1.394	112.6	0.2–0.3
	Ar ^*b*^	−1.0	−1.1	0.0	2.772–2.857	1.613	1.394	112.6	0.0
	Ar ^*b*^	−1.9	14.9	17.1	1.587–5.748	1.546	1.334–1.507	109.0–112.4	29.3
	Kr	−3.0	13.9	17.1	1.587–5.775	1.545	1.334–1.507	109.0–112.4	29.8

^*a*^ Due to the diversity of values occurring in some cases, the minimum and maximum values are shown in the $v_{\min}$–$v_{\max}$ format. ^*b*^ Two stable forms have been found, see text.

## Data Availability

Data available from the author on reasonable request.

## Abbreviations

The following abbreviations are used in this manuscript:

Ng	noble gas
QTAIM	Quantum Theory of Atoms in Molecules
BP	bond path
BCP	bond critical point
MBO	Mayer Bond Order
RMS	root mean square
